# Constantine Lippe

**Published:** 1885-03

**Authors:** 


### CONSTANTINE LIPPE.

At a regular meeting of the Homœopathic Medical Society of the County of New York, held January 14th, 1885, the following resolutions were read and adopted :

"WHEREAS, It has pleased Almighty God in His mysterious providence to remove from his devoted family, from his large circle of trusting patients, and from his professional brethren, Dr. Constantine Lippe, of this city, a member of this Society, who was distinguished alike for his bravery as a soldier and for his skill as a physician ; and

"WHEREAS, It is due to the memory of the deceased that this Society shall bear testimony to his personal and professional worth, and mingle its sorrow on the occasion of his decease with those of his more intimate personal friends and those of his family ; therefore, be it

"*Resolved*, That while this Society bows in humble submission and reverence before its Heavenly Father, who hath thus taken from it one of its most respected members, it also bears willing testimony not only to the careful training which had so admirably fitted the lamented deceased for the arduous labors and the great responsibilities of his profession, and to the admirable result of that training, which was seen in his unusual knowledge of the delicate intricacies of the materia medica and in the great success which attended his professional labors, but also to the manliness of his manhood, on the field of battle, in the social circle, and in his profession, and to his great moral worth in all the relations of his life.

"*Resolved*, That this Society respectfully extends to the devoted widow, to the venerable father, and to the other members of the family of the deceased its earnest sympathy in their great sorrow, humbly trusting, at the same time, that He who hath taken from them a husband, a son, and a brother, will also graciously extend to each of them His heavenly support and comfort.

"*Resolved*, That copies of these resolutions, duly attested by the Secretary, be sent by him to the widow of the deceased and to his venerable and distinguished father, be spread on the minutes, and that they also be sent to the medical journals of New York and Philadelphia for publication.

"A. B. NORTON, M. D.,
"*Secretary*."

